# Organ-on-a-Chip and Microfluidic Platforms for Oncology in the UK

**DOI:** 10.3390/cancers15030635

**Published:** 2023-01-19

**Authors:** Joanne Nolan, Oliver M. T. Pearce, Hazel R. C. Screen, Martin M. Knight, Stefaan W. Verbruggen

**Affiliations:** 1 Centre for Bioengineering, School of Engineering and Materials Science, Queen Mary University of London, London E1 4NS, UK; 2 Centre for Predictive In Vitro Models, Queen Mary University of London, London E1 4NS, UK; 3 Barts Cancer Institute, School of Medicine and Dentistry, Queen Mary University of London, London E1 2AD, UK; 4 Department of Mechanical Engineering, INSIGNEO Institute for In Silico Medicine, University of Sheffield, Sheffield S1 3JD, UK

**Keywords:** organ-on-chip, microfluidic, microphysiological system, pre-clinical model, cancer, oncology, tumour cell, spheroid, mechanobiology

## Abstract

**Simple Summary:**

Organ-on-a-chip models, or organ chips, are tiny devices designed to accurately recreate the natural physiology and mechanical forces that cells experience in the human body. Similar to computer microchips, though carrying fluid through channels instead of an electric current, organ-chips are lined with living human cells and their tiny channels can reproduce blood and/or airflow just as in the human body. Their flexibility allows the chips to recreate breathing motions or undergo muscle contractions. This new and rapidly expanding field of research provides a unique opportunity to build models of human organs and to study how cancer cells develop and spread within them. As these chips are simpler and cheaper than animal models of cancer, they could significantly increase the speed of drug discovery and testing in cancer research. This exciting potential has led to the rapid development of this technology in the United Kingdom, with active research on a range of cancer types. This review covers the broad sweep of organ-chip research in the UK, and the network of researchers and companies being developed. Finally, it concludes with a perspective on the future directions in the field as researchers aim to bring about a leap forward in cancer therapies.

**Abstract:**

Organ-on-chip systems are capable of replicating complex tissue structures and physiological phenomena. The fine control of biochemical and biomechanical cues within these microphysiological systems provides opportunities for cancer researchers to build complex models of the tumour microenvironment. Interest in applying organ chips to investigate mechanisms such as metastatsis and to test therapeutics has grown rapidly, and this review draws together the published research using these microfluidic platforms to study cancer. We focus on both in-house systems and commercial platforms being used in the UK for fundamental discovery science and therapeutics testing. We cover the wide variety of cancers being investigated, ranging from common carcinomas to rare sarcomas, as well as secondary cancers. We also cover the broad sweep of different matrix microenvironments, physiological mechanical stimuli and immunological effects being replicated in these models. We examine microfluidic models specifically, rather than organoids or complex tissue or cell co-cultures, which have been reviewed elsewhere. However, there is increasing interest in incorporating organoids, spheroids and other tissue cultures into microfluidic organ chips and this overlap is included. Our review includes a commentary on cancer organ-chip models being developed and used in the UK, including work conducted by members of the UK Organ-on-a-Chip Technologies Network. We conclude with a reflection on the likely future of this rapidly expanding field of oncological research.

## 1. Introduction

The transition in cancer research from basic fundamental research to drug discovery most commonly involves the culturing of cancer cells in plastic 2D cell culture dishes alongside follow-up studies conducted on animal models. Indeed, at present, compelling preclinical evidence is a pre-requisite for progression into clinical trials [1]. However, given the recent passing of the 2022 FDA Moderinization Act 2.0 by the United States Congress [2,3], this landscape is changing and will provide drug sponsors with the capacity to use alternative complex in vitro models where suitable.

Most animal studies involve the treatment of subcutaneously implanted tumours in rodents. However, it is now well established that these studies are poor representations of human biology, notably because they lack key features of the native tissue microenvironments and mix human and non-human cells [4]. It has been demonstrated that tissue-specific conditions are better approximated by implanting human tumour xenografts in mice at the relevant organ site, with these orthotopic cancer models showing improved recapitulation of both tumour growth and metastasis [4,5]. However, orthotopic mouse models can still not mimic the tumour microenvironment (TME) present in human disease that regulates cancer development and progression, and thus remain limited in their human-relevance. This is particularly pertinent with regard to the immune system, as orthotopic mice are usually immunocompromised to prevent the rejection of the human cancer cells. This significant disadvantage is in ever-greater discordance with our growing understanding of the key role of the immune system in cancer progression and with the rising interest in the use of immunotherapy in oncological research [6].

Collectively, irrespective of the type of animal model used, preclinical studies are broadly accepted to be poor indicators of future therapeutic responses in human clinical trials [1]. This problem is further compounded by the fact that, when investigating physiological interactions and cellular signalling, preclinical studies typically occur at a single timepoint upon termination. In particular, it is extremely difficult to monitor fluctuations in the TME (e.g., immune cells, fibroblasts and stem cells) when looking to identify how elements of the TME influence outcomes. Taken together, the above challenges have stimulated interest in developing more complex, yet easily manipulated, in vitro models of human cancer to accelerate fundamental discovery science and therapeutic testing.

Traditional in vitro cell culturing involves 2D monolayers of cells grown on tissue-culture-treated glass or hard plastic, and has been used for over 50 years to assess the effects of drugs on tumour cell growth [6]. Although these methods gave rise to successful therapies such as the chemotherapies still used in clinics today, these environments cannot replicate the complex multi-cellular environments found in vivo and bear little resemblance to human or animal physiology [7]. This represents a major disadvantage of these flat uniform surfaces, particularly when investigating precision medicine and targeted approaches to improve patient outcomes [8]. All cell types are highly sensitive to changes in their microenvironments, which in vivo comprise a dynamic array of molecular cues from the surrounding matrix, nearby cells, mechanical stimulation and chemokine concentration gradients. While traditional cell culture methods are sufficient for the rudimentary testing of mechanisms and drugs on single cell types, the complete absence of this rich array of mechanical and biochemical cues is a significant limitation to clinical translation [9]. Slightly increased complexity can be generated using transwell-assays or Boyden chambers, which allow the study of the migration and invasion of tumour cells through micro-scale membrane pores. This can be further improved by adding thin layers of substrate (e.g., fibronectin or collagen), thick ECM gels (e.g., Matrigel) [10] or using a confluent monolayer of endothelial cells to model trans-endothelial intra/extravasation. Spheroids, which consist of cells grown in 3D matrix, have also been developed in culture wells, allowing the study of cell–cell or cell–ECM interactions between cancer cells and the surrounding TME, and these can also be made large enough to generate a necrotic core, which is emblematic of poorly vascularised tumours [11,12,13]. However, unfortunately, transwell and spheroid models can neither recreate the complex 3D tissue architecture found in living organs nor introduce mechanical stimulation (e.g., substrate strain, fluid shear stress and hydrostatic pressure), which have been shown to be crucial in regulating tumour cell behaviour [14,15,16,17,18]. Additionally, the static culture conditions present in these wells bear little resemblance to the perfusion by blood or nutrient-rich interstitial fluid that occurs in vivo, precluding the investigation of metastatic mechanisms, immune cell recruitment or physiologically-relevant drug dosing [6].

A recent development in cancer research is the field of organoid culture, wherein samples of normal or cancerous stem cells are isolated from human epithelium and grown in ECM gels. In these conditions, organotypic microstructures self-form, recreating some elements of the TME. These outcomes have led to significant excitement regarding the propsect of a new in vitro tool for both drug discovery and personalised medicine [19]. However, these models still have limitations, the principal of which being that they form closed structures that prevent direct experimental access to the lumen formed by the epithelium. They also lack the tissue–tissue borders between tumour cells and the vasculature and stroma of the matrix, which normally envelop the tumour and are key regulators of cancer progression. Furthermore, a similar major shortcoming is the complete lack of the relevant mechanical stimulation that all cells experience, albeit to varying degrees, within organs and tissues, and which is known to affect tumour cell development and behaviour. These limitations can be overcome using microfluidic approaches, developed in the burgeoning field of organ-on-a-chip reseach.

This review focuses on microfluidic organ-chip devices and the application of novel tools to facilitate oncology research in the United Kingdom. We focus on both in-house systems and commercial platforms being used in the UK for fundamental discovery science and therapeutics testing. We cover the wide variety of cancers being investigated (e.g., common carcinomas and blood cancers, rare sarcomas and secondary metastatic cancers) and the broad sweep of different matrix microenvironments, physiological mechanical stimuli and immunological effects being replicated in these models. The focus is on microfluidic models specifically, rather than organoids or complex tissue or cell co-cultures. However, in cases where these cultures are being deployed innovatively in microfluidic devices, they are discussed here. In this review, we provide a commentary on cancer organ-chip models being developed and used in the UK, including work conducted by members of the UK Organ-on-a-Chip Technologies Network. We conclude with reflection on the likely future of this rapidly expanding field.

## 2. Organ-Chip Technology

Organ chips build upon the established field of microfluids, allowing the development of small-scale devices that can be finely-tuned for cell culture conditions. These chips are fabricated from optically clear plastic, glass or flexible polymers, with a common choice being polydimethylsiloxane (PDMS), to contain microchannels which can house selected populations of living cells and culture media. It should be noted that depsite its widespread use, an inherent limitation of PDMS is the tendency for drugs to be absorbed onto its surface. This could reduce the cell uptake of candidate drugs and is therefore a key challenge for the field. These channels and cavities can be arranged to mimic the in vivo physiology and pathophysiology of organs through building tissue- or organ-level structures in vitro [20]. The term “chips” is a convenient shorthand umbrella term for these microphysiological systems (MPSs), which originates in the microfabrication methods used for their production, which were adapted from those applied in the manufacturing of microchips [21]. The standard FDA definition of an MPS is a microscale cell culture platform for in vitro modeling of functional features of a specific tissue or organ of human or animal origin by exposing cells to a microenvironment that mimics the physiological aspects important for their function or pathophysiological condition. Organ chips are considered a subset of these MPS, and are defined as a miniaturised physiological environment engineered to yield and/or analyse functional tissue units capable of modeling specified/targeted organ-level responses.

These microfluidic devices have been used to create a range of different models, from “tissue chips”, which comprise a single channel and one cell type, through to complex “organ chips” that incorporate multiple cell types representing numerous tissues. These more complex chips are designed to allow communication and crosstalk across porous membranes or ECM barriers, often coated with a type of ECM hydrogel. Steady perfusion of the culture medium at low flow rates through the channels allows cells to be maintained and observed for long time-periods (up to weeks or months, depending on the contact inhibition of the cell type) [20]. Indeed, a recent review by researchers at the University of Oxford has highlighted the importance of both dynamic and long-term cultures for the accurate preservation of human tissue and the replication of human physiology in vitro [22], pointing to the importance of this key strength of organ-chip models. Some organ chips are designed specifically to include an endothelialised channel, which is representative of vasculature, and which can then be used to perfuse suspended immune or cancer cells, or even whole blood [23,24]. The early developments related to these chips have been reviewed elsewhere [6], whereas the fabrication methods involved have been reviewed in detail by researchers at the University of Hull in the UK [25].

### Mechanobiology in Organ Chips

A key advantage of many organ-chip platforms is the ability to simultaneously apply and control multiple biochemical and biomechanical cues to direct cell behaviour, allowing the replication of multiple critical aspects of tissue and organ physiology [20]. It has been well established that biomechanical stimuli influence the growth and formation of almost all tissues in the human body, with these cues known to be modulators of cell signalling in both healthy and diseased environments [26]. As well as affecting the local environment, biomechanical cues can be sensed by cells and transduced to alter cell behaviour, cytokine signalling and gene expression. This field of research, termed mechanobiology, is an expanding area of multidisciplinary research with important implications in regulating cancer cell behaviour. The field of microfluidics presented itself as an ideal way for bioengineers to manipulate cell mechanobiology [27], whereas the innovations involved in organ chip design have produced novel approaches to building organotypic mechanical signals in vitro [28]. Mechanical stimulation is now thought to be a crucial element of organ-chip models, and the standardisation of biomechanical cues is of critical importance when developing these systems (reviewed elsewhere by researchers at Queen Mary University of London [29]). The unique opportunities provided by organ chips to manipulate cancer cell mechanobiology has been highlighted for some specific cancers [30], but will be discussed here in general terms.

## 3. Cancer Phenomena Modelled Using Organ Chips

**Figure 1:**
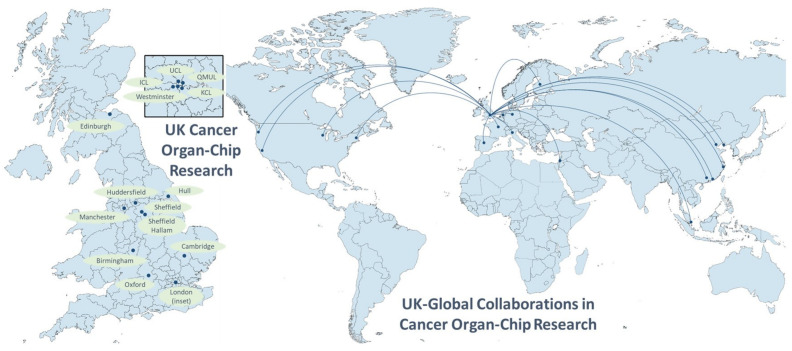
UK cancer research centres building organ-chip models, and worldwide UK collaborations in the field. Global collaborators include institutions in China (Beijing, Dalian, Guangzhou, Hong Kong, Zhejiang), Europe (Finland, France, Germany, Italy, the Netherlands, Spain) and the United States (California, Massachusetts, Washington, Wisconsin).

The rapidly developing field of organ-chip research has been enthusiastically embraced by researchers in the UK and their collaborators across the globe (see Figure 1). Both commercially available systems and custom-designed in-house chips are being used to tackle a wide range of challenges across the breadth of oncological research.

### 3.1. Tumour Growth/Proliferation

**Figure 2:**
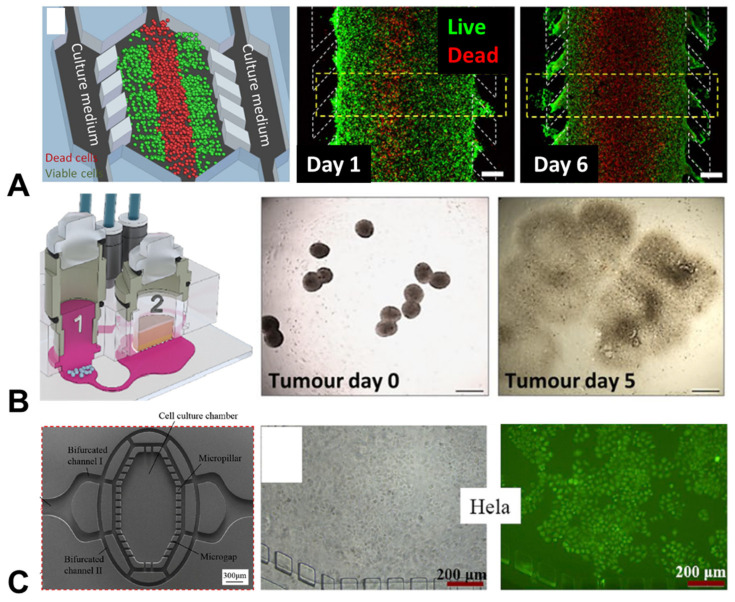
Organ-chip models of tumour growth and cancer cell proliferation. (**A**) Proliferation of cancer cells over 5 days in a chip model of human colon carcinoma (red = dead cells; green = live cells; scale bar = 400 µm) [31]; (**B**) A chip co-culture used to investigate the growth of (1) lung tumours and (2) skin microtissues over 5 days (scale bar = 500 µm) [32]; (**C**) A chip model exploring the proliferation of cervical cancer cells over 5 days, with staining delivered via tumour targetting nanoparticles [33]. Figures reproducued with permission.

A standard measure of tumour growth when using traditional cancer-cell monolayer cultures is proliferation across a 2D surface. Similarly, in the more complex 3D environments that can be developed when using organ chips, the proliferation of cancer cells is a key measure of investigation. A collaboration between researchers at the University of Huddersfield and groups in Spain has taken a broad approach to investigating proliferation, building generalised models of the tumour microenvironments of both colon cancer and glioblastoma (an aggressive form of brain cancer) (Figure 2). These cancer types were specifically selected, as it is currently challenging to build 3D structures (e.g., spheroids) with these cell types. Using a custom-built polystyrene device, the researchers were able to recapitulate many aspects of the tumour microenvironment, demonstrating differences in the rates of cancer cell proliferation, glucose uptake and oxygen consumption, as well the presence of necrotic regions, along with a build-up of damaging reactive oxygen species (ROS) [31].

A separate study, resulting from a collaboration between researchers in Germany and the University of Manchester, integrated proliferation measures as part of the assessment of safety and efficacy of an antibody immunotherapy treatment for a type of lung cancer that had potential side-effects on skin tissue. The group built a multi-organ microfluidic model of the human lung cancer microtissues, alongside a compartment containing human full-thickness skin equivalents. Using this model they were able to demonstrate disruption of both lung cancer cell growth rates and the normal proliferation of skin cells following antibody treatment. This demonstrated the potential of these multi-organ, microtumour models regarding the use of patient-derived cells and tissues for drug discovery, alongside the safety profiling of drug candidates [32].

Separately, a multi-tumour chip model arose from a collaboration between researchers at University College London (UCL) and colleagues in China, containing both cervical and lung cancer cells, with a custom chip design guided by numerical modelling. This model was used to demonstrate that tumour cells could be tracked using fluorescent nanoparticles, allowing live cell tracking and the surveillance of cellular proliferation and tumour growth. Notably, these nanoparticles were found to behave similarly in mouse models, suggesting that this platform represents a useful microphysiological system for modelling in vivo tumour growth [33].

Demonstrating the interest of industry in this area, CN Bio Innovations Ltd. and Astrazeneca PLC, both based in the UK, contributed to a project with US research institutes to build chip models of pharangyeal and lung cancers, with the aim of rigorously matching their growth profiles to in vivo data. As well as succeeding in this, the models managed to replicate the expected pharmacokinetic (PK) responses of the cells to different drugs over time, analogously to in vivo responses. This also represented an important step towards standardised, clinically-relevant outputs from chip models of tumour growth [34].

### 3.2. Modelling the Metastatic Cascade

The cascade of events involved in the metastasis of primary tumours to distant sites is poorly understood, but is universally associated with poorer clinical outcomes in cancer patients. Due to these poor prognoses, metastasis is an area of extreme interest in the field of oncology [35]. Regardless of the primary tumour type, metastasis is a complex, multistep process. Initiated by cancer cells at the primary site, these cells develop the ability to disseminate and successfully invade through the associated basement membrane. After migrating through the interstitial matrix, cells must then undergo transendothelial migration, comprised of intravasation into a vessel, for example, a capillary or lymphatic vessel, permitting the transportation of these cells throughout the body via the circulatory system. To enable this migration, metastatic cells must survive the high shear stresses and pressure changes in the vessels during circulation, evade the immune cells that patrol these vessels and then attach to the endothelium at a secondary site. To establish a metastatic colony, they must then begin the process of extravasation from the vessel and implantation in the tissue of a new organ, which is often a very different microenvironment when compared to the primary site.

This cascade is composed of a number of complex steps, which have not been well captured in monolayer cell cultures. However, the tunability of organ-chip devices has proven useful for building complex 3D microenvironments that represent cancer cells disseminating into the vasculature, as well as extravasating into the tissue.

A research group at the University of Cambridge fabricated a custom-designed PDMS chip to investigate the transendothelial migration of individual breast cancer cells. This platform enabled them to capture the dynamic process of single breast cancer cells exiting the vessel lumen into the surrounding extracellular matrix. Interestingly, the presence of an endothelial lining significantly reduced the cancer cell extravasation events over a 15 h imaging period. A particular strength of the organ-chip approach used here was the ability to map the z-position of individual cancer cells within a 3D vessel representing the lumen, enabling the identification of cancer cell transmigration ‘hot spots’ in real-time, using live-cell imaging. The findings also suggested that variations in the microvessel qualities may result in two distinct types of cancer transmigration behaviour, potentially explaining the success or failure of circulating tumour cells at distant cancer sites [36].

A similar approach was taken in a collaboration between UCL and researchers in Germany, in which custom designed PET-PDMS models of extravasation were built. The team focused on lung and skin cancers, and found that the rapid breakthrough of an epithelial cell layer by cancer cells could be replicated in the chip. Additionally, the researchers were able to manipulate the flow conditions within the vascular channel, discovering that the number of adherent and invading cells depended on both the flow magnitude and flow dynamics (i.e., continuous or pulsatile). The data not only indicated a role for mechanobiology in the metastatic cascade, but also highlighted how organ-chip models may help to explore these processes [37].

Another type of multi-organ chip, developed at Queen Mary University of London, aimed to determine the interaction between breast or prostate cancer cells and osteocytes, the most abundant bone cell type [38]. The use of the PDMS-based Emulate chip system allowed for investigations to be conducted into the effect of mechanical loading in the form of fluid shear, replicating interstitial fluid flow, which is known to play a crucial role in bone mechanobiology and homeostasis in the bone marrow environment. This study showed that the mechanical stimulation of bone cells encouraged the invasion of breast and prostate cancer cells in a chip microenvironment. This may partially explain the difference in the clinical presentation of metastatic lesions between these two types of cancer, and demonstrates advantages of chips over traditional monolayer cultures in mechanobiological studies [38].

These mechanical stimulation techniques may also be applied to spheroid cultures, as performed by researchers at the University of Hull investigating the effect of flow on both glioblastoma and breast cancer cells. By custom-designing a glass microfluidic chip capable of containing singular cancer cell spheroids under continuous flow, they measured the increased release of pro-metastatic factors and inflammatory cytokines when stimulated mechanically. This suggests that the inflammatory response of cancer cells can be modulated by mechanical stimulation, which may have potential implications when considering interactions between cancer cells and immune cells [39].

**Figure 3:**
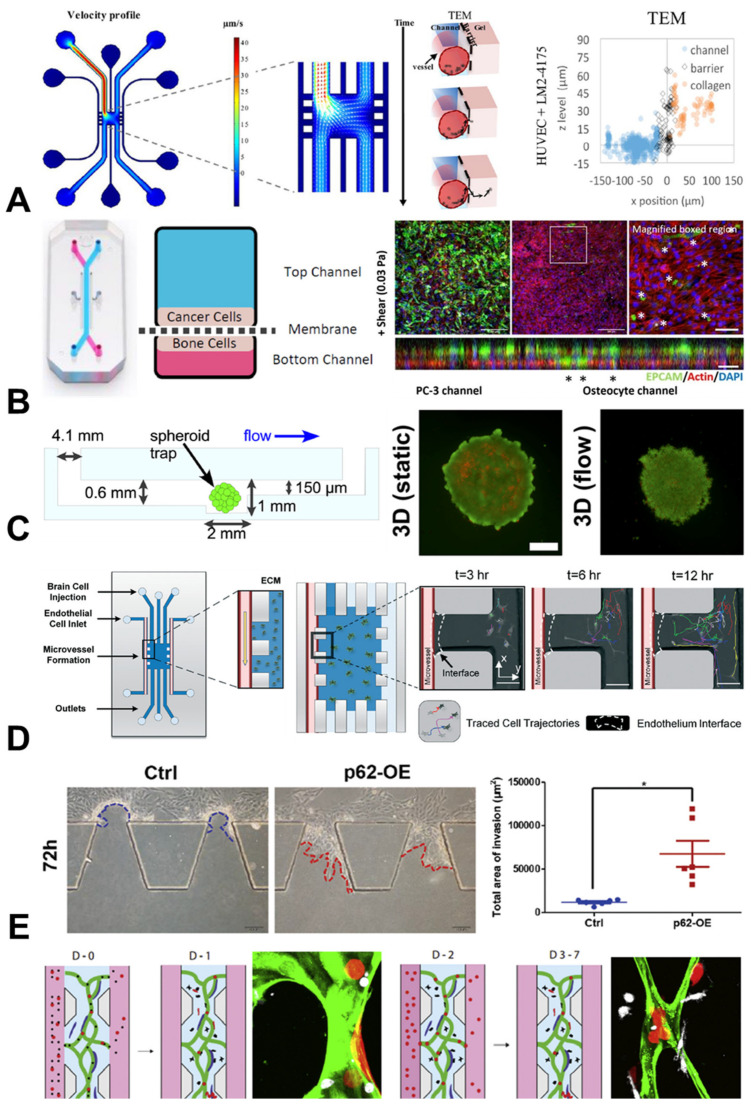
Organ-chip models investigating the metastatic cascade. (**A**) A chip model investigating the effect of fluid flow and barrier formation on the transendothelial migration (TEM) of breast cancer cells [36]. (**B**) A mechanobiological model showing increased invasion of breast and prostate cancer cells when co-cultured in a chip with mechanically-stimulated osteocytes (scale bar = 20 µm; * indicates instance of invasion) [38]. (**C**) Live/dead staining of breast cancer spheroids, showing more growth under flow conditions when compared to static conditions (scale bar = 200 µm) [39]. (**D**) Live-cell tracking of migration trajectories of glioblastoma cells over 12 h in a chip with microvessels, crossing an epithelial barrier and entering the extracellular matrix (ECM) (scale bar = 100 µm) [41]. (**E**) Increased invasion of breast cancer cells in a chip when over-expressing the p62 gene (p62-OE) (scale bar = 100 µm; * indicates *p* < 0.05, two-tailed Student’s *t*-test) [42]. (**F**) The rate of breast cancer cell (red) extravasation from microvessels (green) over seven days (D-0 to D-7) was affected by the presence of monocytes (white) (scale bar = 10 µm) [43]. Figures reproduced with permission.

The impact of mechanical stimulatin on cancer and immun cell interactions has been investigated by researchers in the University of Birmingham, in collaboration with colleagues in Singapore and Boston, using a combined in vitro and computational approach to model macrophage migration under the influence of cancer cells. In a complex 3D organ-chip model, the team observed that macrophage migration was influenced by the combined presence of tumour cells and interstitial fluid flow. Building upon this in vitro work, the researchers built an in silico model to link these observed behaviours to cytokine and mechanosensitive signalling (Figure 3). Most interestingly, the researchers found that, although immune signalling from tumour cells could dampen the macrophage response via cytokines such as IL8 or CCL2, mechanical stimulation of immune cells could override this inhibition. This highlighted the importance of integrating mechanobiology into immunotherapy-directed research, as well as demonstrating how computational techniques can add greater insights when coupled with organ-chip technologies [40].

Glioblastoma cells must develop the ability to control the perivascular niche in order to manipulate its local microenvironment. This niche is particularly difficult to replicate in either animal models or traditional cell cultures; therefore, organotypic chip models may be of particular benefit to exploring this niche. With an organ chip built to a custom design from PDMS and containing endothelial cells from a range of different human origins (brain microvasculature, umbilical vein and lung microvasculature), researchers in Cambridge, Edinburgh and London collaborated to investigate whether the vascular environment affected the behaviour of glioblastoma cells. Endpoint biological assays, live cell imaging and qPCR showed that glioblastoma cells behaved in a more organotypic fashion when co-cultured within a perivascular niche comprising cells of brain origin. They also found that in these endothelial cells, genes for neovascularisation were upregulated, suggesting cancer cell manipulation of this environment [41].

As well as exploring the broad behavioural phenotypes of cancer cells, organ chips can also be used as fundamental science tools to target specific genes and study the resulting interactions. A team from Imperial College London (ICL) worked with colleagues in China to build a custom-designed PDMS organ chip to investigate p62 expression in triple-negative breast cancer cells [42]. Through a series of chip experiments, the researchers observed that the genetic ablation of p62 reduced the development of invasive protrusions within the cancer cells. Building upon this, mass spectrometry was used to reveal strong interactions between p62 and vimentin, with further experiments confirming this link and providing a new therapeutic avenue of scientific enquiry for this difficult-to-treat breast cancer subtype.

Another interesting collaboration exploring immune–vascular interactions using organ chips was established between researchers at the University of Sheffield and US colleagues in California, Seattle and Boston. The groups showed evidence of direct engagement between triple-negative breast cancer cells and monocytes in a 3D vascular niche, finding that this engagement affected the differentiation of monocytes into macrophages. They also found a reciprocal role for the monocyte/macrophages in controlling the degree of migration and intra/extravasation that was possible in the vascular environment. This demonstrated a complex cell-crosstalk at play within this chip microenvironment, which could be further exploited to unpick the molecular mechanisms involved [43].

### 3.3. Cancer-Associated Behaviours

As well as the wide-ranging possibilities provided by organ chips to measure the direct interactions between cancer cells and tissue-relevant cell types, these tuneable microenvironments grant the possibility to understand fundamental mechanisms in health and development. Understanding these mechanisms is critical for comprehending how their disruption is implicated in malignancy. The behaviour and differentiation of cells in epithelial layers under various types of mechanical stress is a crucial area of study in development, with cell fate known to be connected with forces experienced across an epithelium. This is of importance for cancer research, as the epithelium is the overriding source of malignancy in many major cancers. Furthermore, a major step in the progression of many cancers is the disruption of the epithelial barrier, which is associated with the growth and spread of the disease.

**Figure 4:**
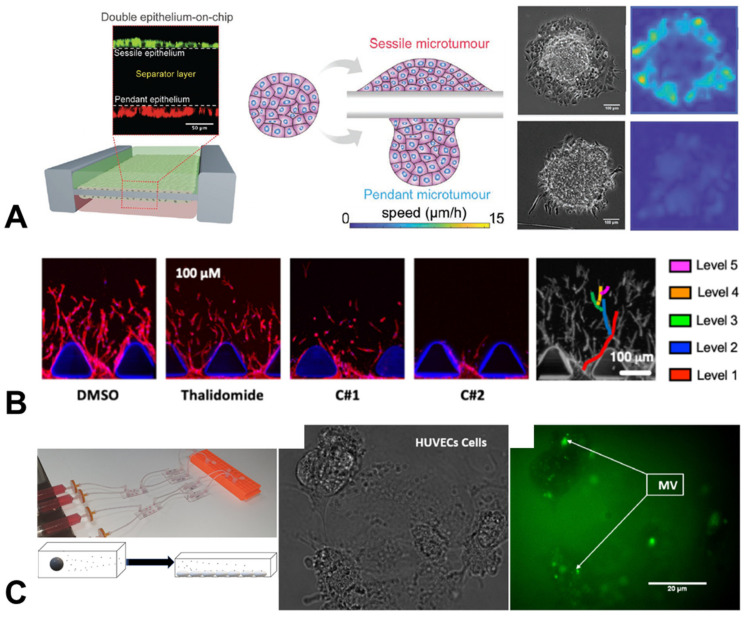
Organ-chip models of cancer-associated behaviours. (**A**) The spreading speed of liver microtumour spheroids depended on the epithelial layer on which they are placed (scale bar = 100 µm) [44]. (**B**) Angiogenic sprouting, modelled and analysed in a microfluidic chip under a range of different anti-angiogenic drug treatments (thalidomide, C#1, C#2) (scale bar = 100 µm) [45]. (**C**) Chips placed in series allowed the monitoring of the uptake of procoagulant microvesicles secreted by glioblastoma and ovarian carcinoma cells (MV, green) by human endothelial cells (scale bar = 200 µm) [46]. Figures reproduced with permission.

Investigating this behaviour, collaborators from Kings College London (KCL), Singapore, Israel and China built an organ chip that replicated the liver epithelium (Figure 4). Using RNA sequencing [44] and transcriptome analysis, they linked RhoA, BMP2 and hypoxia-related genes with microtumour-spreading behaviour and the onset of the epithelial-to-mesenchymal transition, a key step required for most cancer cells to become metastatic. This study demonstrated how chips can be used to probe the biophysical principles underlying the metastatic cascade, as well as broader cancer cell behaviours.

Another key event required for tumour progression is angiogenesis, as growing tumours require ever-increasing nutrient supplies, beyond what is available in their initial microenvironment. Additionally, the vasculature is also a common route for metastatic cells to access distant sites, and thus angiogenesis presents a tantalising target for therapeutic intervention to improve patient prognoses. Researchers at the University of Sheffield, alongside colleagues in Italy, Singapore and Boston, built a 3D microvasculature environment in order to assess the effects of various anti-angiogenic agents on the development of new blood vessels in cancer [45]. This chip used human cells, cross-validated against an in vivo zebrafish model, enabling the team to rapidly screen for the most effective anti-angiogenic compound, while also testing effects on cancer cells and monitoring toxicity. This study demonstrated how organ chip devices can simultaneously be applied to fundamental discovery science and therapeutic testing, a theme which will be expanded on in the final section.

An interesting cancer-associated behaviour that has been observed clinically in a range of cancer types is increased coagulation or clotting in blood. It is not entirely clear why this occurs, with theories ranging from an increased inflammatory response to actively aiding metastatic spread via attachment to the endothelium. This is extremely difficult to study in vivo, and chips present an ideal testbed to monitor the effect of circulating signalling molecules and microvesicles released by cancer cells into conditioned media. A group at the University of Hull took this exact approach, observing the upregulation of procoagulation activity in human endothelial cell chips when exposed to conditioned media from pancreatic, ovarian and glioblastoma cancer cells [46,47].

### 3.4. Modelling Responses to Cancer Treatments

While the use of organ chips to completely replace animals in preclinical testing is a widely-held hope for the field, perhaps the most exciting and more immediate prospect for organ chips lies in their ability to rapidly screen for leading drug candidates. Nowhere is this problem more pressing than in the testing of new cancer drugs, as oncological treatments have the lowest success rate of any therapeutic field, with only 5.1% of cancer drugs that enter Phase I clinical trials ultimately being approved by the FDA [48]. Even with promising pre-clinical data, bringing a single drug to FDA market-approval can take more than 10 years and USD 2.5 billion, with about two-thirds of this cost occurring in the clinical trial phases [49]. By using multiple human cell types to recapitulate in vivo microenvironments and cell crosstalk, organ chips provide the means to exclude less promising candidate therapies before clinical trials, freeing up funding to pursue new avenues of enquiry.

Examples of this in action in the UK have been mentioned above, with researchers in Huddersfield testing the reaction of colon cancer and glioblastoma cells under doxorubicin treatment [31]. Similarly, researchers in Manchester tested the effectiveness of an anti-EGFR antibody immunotherapy treatment, while also testing for side-effects in healthy skin cells, describing their work as safficacy testing (safety and efficacy simultaneously) [32]. As described in the previous section, chips can also be used to test for the effects of drugs on cancer-associated behaviour, such as the work carried out by researchers in Sheffield to test for candidates similar to thalidomide that could reduce angiogenesis, while also screening for toxicity before testing in zebrafish [36].

Immunotherapy testing is of key interest in cancer, and researchers at UCL have worked with international partners to construct a 3D microfluidic microenvironment using SU-8 materials [50]. This breast tumour model provided an excellent testbed for immune interventions, with the authors measuring the effects of TNF-related apoptosis inducing ligand (TRAIL) on tumour growth and viability. Interestingly, they found that combining TRAIL with a vesicle-based delivery system could significantly increase the uptake and efficacy of the treatment, suggesting that chips provide an excellent system for honing the effectiveness of controlled delivery techniques [50].

**Figure 5:**
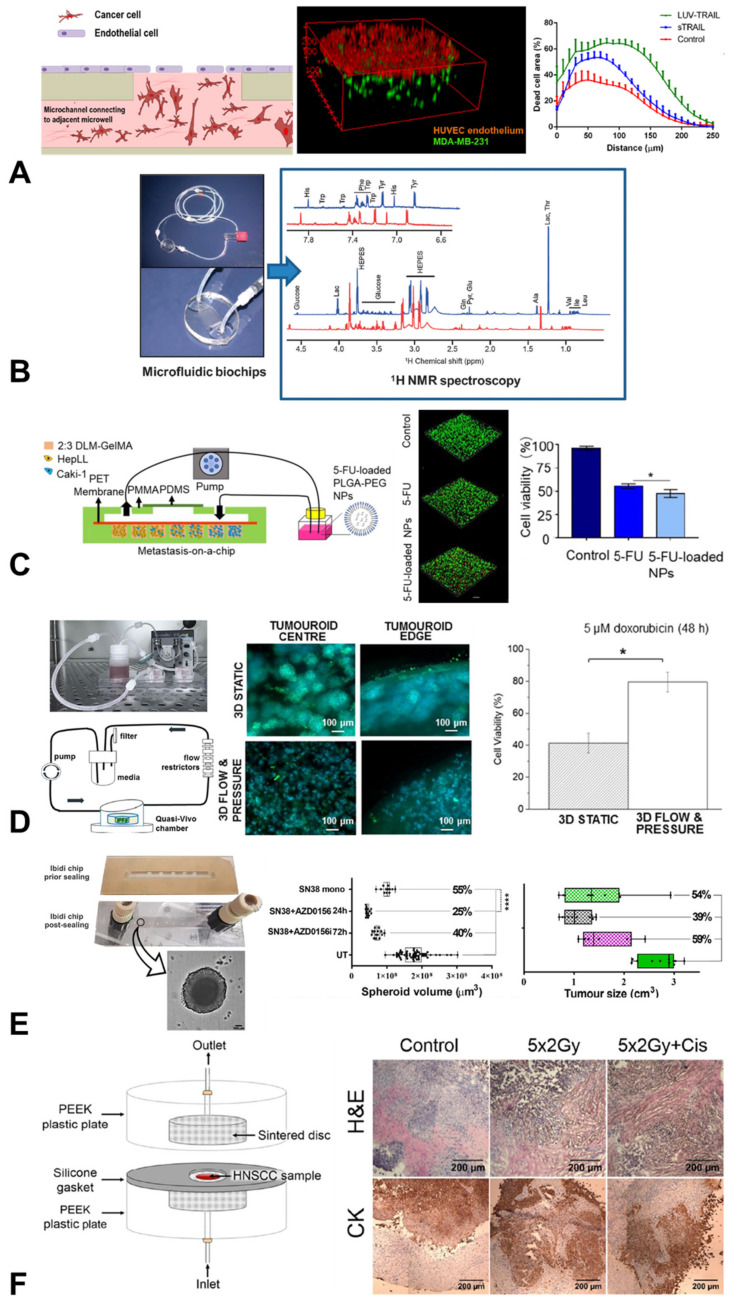
Organ-chip models applied for therapeutic testing in oncological research. (**A**) A chip model to test the delivery of TRAIL treatment to breast tumour cells [50]. (**B**) Organ-chip models of liver cacinoma can be applied to build metablomics profiles under a range of drug treatments [51]. (**C**) Testing of enhanced drug delivery via nanoparticles to kidney cancer cells (scale bar = 100 µm; * indicates *p* < 0.05, one-way ANOVA) [53]. (**D**) A tumouroid chip model of breast cancer cells, demonstrating increased resistance to doxirubicin treatment under mechanical stimulation (scale bar = 100 µm; * indicates *p* < 0.05, paired Student’s *t*-test) [54]. (**E**) A chip model demonstrating similar response profiles of colorectal cancer spheroids to drug treatments to those observed in implanted tumours in immunocompromised mice (scale bar = 100 µm; **** indicates *p* < 0.0001, one-way ANOVA with Tukey’s post-hoc test) [55]. (**F**) A custom-desgined chip investigating the effect of radiation and cisplatin treatment on tissue samples of head and neck squamous cell carcinomas (scale bar = 200 µm) [56]. Figures reproduced with permission.

A similar approach taken by researchers at ICL, alongside colleagues in France, led to the development of a “metabolomics chip” [51], in which cells could be cultured while providing readouts of metabolites from the culture. This was developed using liver cancer cells, which importantly allowed the testing of the drug compounds for toxicity. One application of this was in testing flutamide, an anticancer drug, as well as its downstream metabolite, hydroxyflutamide (Figure 5). This microphysiological system allowed the team to extract mechanisms of action and propose a metabolic network for the activity of the drug, suggesting that this metabolomics chip may provide a useful alternative in vitro method to predictive toxicology [52].

Alongside the liver, the kidney is an organ affected by treatment toxicity, and therefore an important organ to monitor during drug discovery. A team at the University of Cambridge recently built a custom-designed PDMS chip, in collaboration with groups in China, Finland and California, modelling kidney cancer progression to the liver, a common metastatic site for this cancer type. By using a combination of different cells, they were able to predict the treatment efficacy of 5-fluoracil, an anti-cancer drug, on metastatic kidney cells, while also screening for its effects on hepatocytes [53].

Drug testing using organ chips can be augmented to incorporate more complex structures such as spheroids, while still recapitulating some of the mechanical stimulation experienced in vivo. This was the approach taken in a collaboration between the University of Westminster and UCL, in which researchers developed a PDMS in vitro 3D model comprising a central high-density mass of triple-negative breast cancer cells surrounded by collagen type-1 and incorporating fluid flow and pressure [54]. Under these physiological conditions, cells expressed less of a response to doxorubicin treatment, alongside decreased expression of vimentin and the key oncogenes HER2 and Ki67. These data highlight how this microphysiological system may now present a platform on which to readily test drug effectiveness and safety for breast cancers.

A similar spheroid approach was taken by a Cambridge research group to investigate colorectal cancer. The team used a chip model comprising commercially available Ibidi polystyrene chips, adding Matrigel-encapsulated colorectal cancer spheroids to the microfluidic system. This allowed them to test a number of drugs on their experimental model, measuring the pharmacodynamic (PD) response and finding that it matched in vivo mouse plasma exposure profiles well. This spheroid approach also allowed them to monitor spheroid volume and viability, which were analogous to clinical measurements used in monitoring these tumours [55].

The testing of cancer therapeutics is not limited to drug screening. In a collaboration between the University of Hull, the University of Sheffield and Sheffield Hallam University, researchers investigated the effects of radiation treatment on rare head and neck squamous cell carcinomas (HNSCC) using a microfluidic model. The aim of this study was to develop patient-specific prognoses in response to radiation to help refine treatment options for clinicians. Taking patient biopsies and growing them in a glass organ-chip microenvironment, they were able to faithfully demonstrate the significant inter-patient variability seen in the clinic [57,58]. In addition, the research team built another simple microfluidic model using PEEK and sintered disks to hold samples of patient tissues [56], allowing the testing of both thyroid and HNSCC patient tissue resistance to radiation and chemo treatments [59]. This demonstrates the multi-use possibilities of organ chips across the spectrum of current oncological treatments (Table 1), as well as providing new potential therapeutic targets.

## 4. Perspective on Organ Chip Research in UK Cancer Research

The organ-on-a-chip (OOAC) concept emerged about 10 years ago, when scientists combined fluidic systems and analytical methods with both 2D and 3D cell culture protocols into new in vitro models. Since then, the global organ-chip market has expanded exponentially, being recently valued at USD 21 million and projected to reach USD 220 million by 2025 [3]. The majority of these companies reside in the US, UK, the Netherlands, and France, whereas new entrants to the field are emerging in South Korea, Japan and Taiwan. The founding of a Europe-wide organisation dedicated to this young field, the European Organ-on-Chip Society (EUROoCS), is driving further collaboration between these countries. Research to date has largely focused on developing heart, intestine, kidney, liver and lung humanised chips [4].

Historically, an early attempt to gather the key academic and industry stakeholders occurred at the 2014 “t4 transatlantic think tank for toxicology”, which held a workshop focused on the State of the Art in 3D Organ Chip Cultures. This group, which included representation from the UK, released a detailed report outlining the immediate challenges facing the field, including those related to cancer [61]. The recommendations in this report bolstered the effort to build the field by focusing on drug testing as an application with clear value for both the market and regulators.

This activity has also benefited work towards the additional goal of reducing the use of animal models in research and drug testing, with the establishment of centres tasked with sourcing new technologies to improve the efficiency of preclinical testing. This began with the Centre for Alternatives to Animal Testing at Johns Hopkins University, with a satellite location at the University of Konstanz in Germany. The UK followed with the founding of the Animal Replacement Centre of Excellence at Queen Mary University of London, with Canada later launching the Canadian Centre for Alternatives to Animal Methods at the University of Windsor. These dedicated research centres act as nuclei for creating further awareness of the opportunities provided by these technologies.

The progress towards this goal of more physiologically relevant models was addressed at a workshop held in the United Kingdom (the National Centre for the Replacement, Refinement and Reduction of Animals in Research and Medical Research Council Centre for Drug Safety Science, 2018) and included an overview of organ chip technology and its utility. A regulatory view of organ-chip technologies was outlined by David Jones of the UK Medicinces and Healthcare products Regulations Agency (MHRA), noting that in the future organ chips may be applied in human clinical trials to improve safety and efficacy profiles. Furthermore, scientists from the UK were involved in an “Organ on chips: Current gaps and future directions” conference led by GSK and the Biochemical Society (2019). This conference assessed the current challenges involved in the widespread adoption of these model and the design of strategies which may help to surmount these challenges [62]. The UK is at the forefront of engaging with standardisation and regulation matters associated with this field and, although the outlook is promising, it is clear that organ chips require further validation and improved translational understanding. Importantly, the current systems lack endocrine and immune responses, limiting their ability to mimic human physiology in its entirety. Furthermore, efforts to democratise the expensive and resource-consuming organ chip technologies are underway via industrial-focused research companies, such as the Medicines Discovery Catapult (Innovate UK), which aims to facilitate the availability, penetration and impact of these approaches to the UK drug discovery community [63].

As an indication of the growing partnership between industry and academia in this field, in 2020, Kirkstall Ltd., one of the leading organ chip commercial manufacturers in the UK, organised a special meeting of the Advances in Cell and Tissue Culture Conference focussed specifically on the topic “Towards More Predictive, Physiological and Animal-free In Vitro Models”. This was attended by all the major academic and industrial players from the UK and Ireland. The major conclusion of this meeting was that in order to gain acceptance for organ-chip technologies, it is necessary to target specific preclinical or clinical tests to replace, and to tailor different models to replace specific tests. This was deemed to increase the likelihood of success over aiming to replicate entire systems faithfully [64]. This tension between scalability provided by industry and flexibility provided by self-made custom-designed systems was explored in a recent international survey across 35 countries, providing a useful guide for developing the next generation of organ chips [65].

As a measure of the growing interest in this field, in 2018 the UK Research and Innovation (UKRI) funding agency supported the creation the Organ-on-a-Chip Technologies Network, a Technology Touching Life initiative funded by three research councils. This network ran symposia, public engagement events and training sessions and provided seed grants to draw new researchers into the field. The Network also helped to establish the Queen Mary + Emulate Organs-on-Chips Centre at Queen Mary University of London and provided joint proof-of-concept funding for 18 projects using the Emulate platform. The network has played an active role in shaping policy within the United Kingdom, serving as an expert advisor on animal replacement for key stakeholders, including the Royal Society for the Prevention of Cruelty to Animals (RSPCA), the UK Government Home Office and the UKRI Biotechnology and Biological Sciences Research Council (BBSRC). This engagement with policy-makers is key to further developing standardised regulations, alongside partners in the Regulatory Advisory Board of EUROoCS for the European Medicines Agency (EMA).

This Network stimulated a key study from the University of Leeds, which surveyed a broad range of organ-chip developers and end-users [66]. Despite the wide variety of responders, there was a high level of agreement on the technological bottlenecks faced by the community, the requirements for new technologies and services and the need for more detailed validation of individual and interconnected models in order to reach the more advanced technological readiness levels required for broader adoption. Based on the survey, many developers and end-users agreed that organ-chip technology was currently at a low level of technological readiness. This suggests that the field is still far from robust and lacks consistent validation. Thus, there is an urgent need for further advancement of, and clarity in, these testing activities to achieve the broad adoption and acceptance of organ chips by large pharmaceutical companies and regulatory agencies [66]. One noteworthy comment in this study concerned the need for greater awareness of industry standards among academics developing models for research. Specifically, although research groups may choose not to be fully compliant with regulatory and quality standards, their work would benefit from greater familiarity with industry standards for quality management, such as ISO:9001 and ISO:13485. This would ease the transition to the start-up and scale-up phases [66].

Taken together with the meetings and reports already mentioned, the survey results demonstrated that there is much overlapping interest and a willingness within the organ-chip community to co-develop existing platforms, ancillary technologies and tools required for the widespread use of organ chips. These active and collaborative research efforts, in both industry and academia, could accelerate the advancement of organ chips to the market and enable the broader adoption and acceptance of the technology, ultimately continuing to support this current exponential growth of the market in the years to come.

A further key activity of the Organ-on-a-Chip Technologies Network lies in supporting early-career researchers in the field in order to help the sector flourish as it grows. A particular challenge associated with this is the difficulty inherent in changing a chip model once it has been established in a laboratory, particularly as time spent altering a model to fit field standards or regulatory requirements can feel like a backward step in driving a career. Therefore, the Network has identified that the development of standardised protocols, for example, for the reliable alteration of substrate stiffness inside chips, is urgently required so that methods are widely reproducible and findings are repeatable across the field.

As a final note, although the above describes the outlook for organ chips as a whole, perhaps the greatest opportunity for regulatory approval lies in the oncological space. With a projected estimation of one in every two people being diagnosed with cancer in their lifetime, the current timeline for the development of oncology therapeutics is inadequate. The development of cancer treatment remains exorbitantly costly, and it has the lowest success rates in terms of FDA approval. By drawing together the exciting and rapidly developing research in this space in the UK, we hope to spur on these efforts and demonstrate their importance in the development of the both the field and the market.

## 5. Conclusions

It is clear that organ-on-a-chip research presents a unique opportunity to accelerate both fundamental discovery science and therapeutic testing across the broad range of oncological research. By providing organ-chip models that can better mimic human tissues and the tumour microenvironment, new avenues of scientific enquiry are being explored. Similarly, organ chips provide an additional screening step to determine leading drug candidates early, reducing the need for animal testing and concentrating investments on the most promising targets.

This technology has generated significant interest in the United Kingdom, with a growing network of academic and industrial researchers that is now well-established. Strong engagement with regulatory bodies and a focus on standardisation and reproducibility will be key to the future success of this field. Researchers in the UK are focusing on this in the next generation of organ chips, in order to spur the development of new cancer treatments.

## Figures and Tables

**Table 1:** Published organ-on-a-chip studies of cancer carried out by UK institutions and companies, demonstrating the breadth of models being developed in oncology.

Study	Chip System	Chip Material *	Organ/Cancer	Cell Lines/Tissue	Drug Treatment	Mechanical Stimulation
Algarni et al., *Bio-Microfluidics* 2019 [46]	µSlide I Luer, Vena8	PS, COC	Ovarian, Brain	ES-2, U87, HUVECS	-	Flow
Algarni et al., *Thromb. Res*. 2016 [47]	Custom	PS	Pancreas, Brain	ASPC1, U87	-	Flow
Ayuso et al., *Sci. Rep*. 2016 [31]	Custom	PS	Colon, Brain	HCT-116, U-251 MG, Jurkat	Doxorubicin	Flow
Azimi et al., *Sci. Rep*. 2020 [54]	Quasi-vivo	PDMS	Breast	MDA-MB-231, SKBR3	Doxorubicin	Flow
Bertulli et al., *Sci. Rep*. 2018 [36]	Custom	PDMS	Breast	MDA-MB-231, LM2-4175	-	-
Boussommier-Calleja et al., *Biomaterials* 2019 [43]	Custom	PDMS	Breast	MDA-MB-231, MDA-MB-435, HUVECS, Donor monocytes	-	-
Cai et al., *Adv. Mat*. 2019 [44]	GeminiChip	Glass	Liver epithelial	HepG2	-	Compression
Carr et al., *Head Neck Surg*. 2014 [58]	Custom	Glass	Head & Neck	Human Tissue	Radiation	-
Cheah et al., *Int. J. Onc*. 2017 [57]	Custom	Glass	Head & Neck	Human Primary Cells	Radiation	-
Collins et al., *Bio-* *microfluidics* 2021 [39]	Custom	Glass	Brain, Breast	U87 MG, MCF-7	-	Flow
Gerigk et al., *Lab Chip* 2021 [41]	Custom	PDMS	Brain	U87, Primary, HUVECS	-	-
Hübner et al., *Sci. Rep*. 2018 [32]	MOC	PS	Lung	NCI-H292, Skin tissue	Cetuximab	Flow
Kennedy et al., *Sci. Rep*. 2019 [59]	Custom	PEEK, sintered disk	Head & Neck	Human Tissue	Radiation, Cisplatin	-
Kühlbach et al., *Bioengineering* 2018 [37]	Custom	PET, PDMS	Lung, Skin	H838, SK-Mel 28, HPAEC	-	Flow
Lee et al., *Int. Biol*. 2020 [40]	Custom	PDMS	Pancreas	Panc1, hTERT-HPNE	-	Flow
Li et al., *Carcin* 2017 [42]	Custom	PDMS	Breast	MDA-MB-231, SKBR-3, BT549, MCF-7, SUM149, MCF-10A, HEK293T	-	-
Mercurio et al., *Front Pharmacol.* 2019 [45]	AIM Biotech	PS	Angiogenesis	HUVECS	Thalidomide	-
Naumovska et al., *Int. J. Mol. Sci*. 2020 [60]	MIMETAS	PS	Colon	Caco-2, hiPSC	-	Flow
Ouattara et al., *Mol. Biosyst*. 2012 [51]	Custom	PDMS	Liver epithelial	HepG2/C3a	-	-
Petreus et al., *Commun. Biol*. 2021 [55]	Ibidi	PS	Colorectal	SW620	Irinotecan (SN38)	-
Riley et al., *BMC Cancer* 2019 [56]	Custom	PEEK, sintered disk	Thyroid	Human Tissue	-	-
Singh et al., *PLOS Biol.* 2022 [34]	Custom	PDMS	Pharyngeal, Lung	FaDu, Calu-6, A549	-	-
Choucha Snouber et al., *Toxic Sci*. 2013 [52]	Custom	PDMS	Liver epithelial	HepG2/C3a	Flutamide	-
Verbruggen et al., *Cancers* 2021 [38]	Emulate	PDMS	Breast, Prostate, Bone	MDA-MB-231, PC3, MLO-Y4	-	Flow
Virumbrales-Muñoz et al., *Sci. Rep*. 2017 [50]	Custom	SU-8	Breast	MDA-MB-231, HUVECS	TRAIL	-
Wang et al., *Theranostics* 2020 [53]	Custom	PDMS	Kidney, Liver	Caki-1, HepLL	5-Fluorouracil	-
Wei et al., *Talanta* 2021 [33]	Custom	PDMS	Cervical, Lung	HeLa, A549	-	-

Abbreviations: PS = polystyrene; COC = cyclo olefin copolymer; PDMS = polydimethylsiloxane; PEEK = polyether ether ketone; PET = polyethylene terephthalate; TRAIL = TNF-related apoptosis inducing ligand.
